# Identification of tumor-suppressor genes in lung squamous cell carcinoma through integrated bioinformatics analyses

**DOI:** 10.32604/or.2023.030656

**Published:** 2023-11-15

**Authors:** HENG LI, YOUMING LEI, GAOFENG LI, YUNCHAO HUANG

**Affiliations:** 1 The 2nd Department of Thoracic Surgery, The Third Affiliated Hospital of Kunming Medical University, Yunnan Tumor Hospital, Kunming, 650118, China; 2 The 1st Department of Thoracic Surgery, The Third Affiliated Hospital of Kunming Medical University, Yunnan Tumor Hospital, Kunming, 650118, China; 3 Department of Geriatric Thoracic Surgery, The First Affiliated Hospital of Kunming Medical University, Kunming, 650032, China

**Keywords:** LUSC, DEGs, WGCNA, PPI network, Tumor-suppressor

## Abstract

Lung cancer is a prevalent malignancy, and fatalities of the disease exceed 400,000 cases worldwide. Lung squamous cell carcinoma (LUSC) has been recognized as the most common pathological form of lung cancer. The comprehensive understanding of molecular features related to LUSC progression has great significance in LUSC prognosis assessment and clinical management. In this study, we aim to identify a panel of signature genes closely associated with LUSC, which can provide novel insights into the progression of LUSC. Gene expression profiles were retrieved from public resources including gene expression omnibus (GEO) and the cancer genome atlas (TCGA) database. Differentially expressed genes (DEGs) between LUSC specimens and normal lung tissues were identified by bioinformatics analyses. A total of 66 DEGs were identified based on two cohorts of data. CytoHubba plugin of Cytoscape software was utilized for the further analyses of the top 10 candidate hub genes including OGN, ABI3BP, MAMDC2, FGF7, FAM107A, SPARCL1, DCN, COL14A1, and MFAP4 and CHRDL1, which showed significant downregulation in LUSC. Two LUSC cell lines were used to validate the functions of CHRDL1 and FAM107A through overexpression experiment. Together, our data revealed novel candidate tumor-suppressor genes in LUSC, suggesting previously unappreciated mechanisms in the progression of LUSC.

## Introduction

There was 19.29 million new cases of cancer diagnoses around the globe in 2021, and 9.96 million people died as a direct result of cancer, among which lung cancer accounts for about 25% of cancer-related fatalities [1]. There are two subtypes of lung cancers: small cell lung cancer (SCLC) and non-small cell lung cancer (NSCLC). Patients suffering from non-small cell type occupy about 85% of the total lung cancer cases [2,3]. Types of NSCLC can be further classified into lung adenocarcinoma (LUAD), lung squamous cell carcinoma (LUSC) and large cell lung cancer (LCLC) [4,5]. Among these subtypes, LUSC is the second most common form in both men and women [6]. There are many treatment approaches for LUSC: surgical resection [7], radiotherapy [8], targeted therapy [9], and chemotherapy [10]. Surgical resection is the most straightforward therapy for LUSC patients in the early stages. However, advanced LUSC can develop drug resistance, and the recurrence of LUSC after surgery and treatment largely compromises the treatment outcome [10].

Previous studies suggested that different subtypes of lung cancers display distinct molecular patterns. For example, it has been reported that LUAD and LUSC showed different transcriptomic changes and distinct molecular interaction networks [11]. In addition, single cell RNA-seq analysis revealed the heterogeneity of the tumor microenvironment in different subtypes of lung cancers [12]. These unique molecular patterns can be used for further diagnostic and prognostic purpose. Different growth patterns and the divergence in drug responses of lung cancer subtypes inform the necessity of tailored therapeutic strategies for optimal treatment outcome [13,14].

Altered transcriptomic landscape is a common feature linked to the progression of different cancers [15,16]. Previous studies mainly concentrated on the elucidation of the driving factors (oncogenes) in lung cancer [17]. However, the occurrence and evolution of cancers are associated with the activation of proto-oncogenes and the ineffective expression of tumor suppressor genes. Our limited knowledge about the tumor suppressor genes in lung cancer is an obstacle to the holistic understanding of cancer progression and the development of novel treatment strategy based on synthetic lethality. The combinatory mutation pattern of oncogenes and tumor suppressor genes can jointly impinge on the prognosis of cancer patients [18]. A recent research also indicates the potential application of tumor suppressor gene profiles for the diagnosis in NSCLC patients [19]. Nevertheless, there is a lack of investigation into the tumor suppressor genes in the LUSC.

In the course of this research, we investigated the microarray data collected from two cohorts of LUSC patients, and analyzed the differentially expressed genes (DEGs) between the LUSC samples and normal lung tissues. We applied functional enrichment and the protein-protein interaction (PPI) network analysis to understand the main biological processes and molecular pathways enriched in the DEGs. We further revealed the top ten hub genes which showed significant downregulation in the LUSC samples. The tumor-suppressive functions of CHRDL1 and FAM107A were validated in two LUSC cell lines. Collectively, our analyses identified novel candidate tumor suppressor genes in LUSC, indicating that the downregulation of these tumor suppressors may underlie the progression of LUSC.

## Materials and Methods

### Data retrieval

The gene expression omnibus (GEO) and the cancer genome atlas (TCGA) databases were called to filter out the expression datasets containing LUSC samples, and the following URLs for each database were used for data mining (http://www.ncbi.nlm.nih.gov/geo) and (https://www.cancer.gov/about-nci/organization/ccg/research/structural-genomics/tcga). The TCGA dataset contains 49 normal samples and 502 LUSC samples. The GSE33479 dataset includes 13 normal samples and 14 LUSC samples.

### DEG analysis

With the aid of the limma tool in the R package, the DEGs between the LUSC specimens and normal lung samples were identified [20]. The average gene expression level was calculated after converging different probe sets. Genes with a fold change (FC) exceeding two and the adjusted *p* value less than 0.05 were considered as DEGs.

### Weighted correlation network analysis (WGCNA)

WGCNA package [21] was used to systematically characterize the gene association patterns of various samples. Highly coordinated gene sets were identified based on the associations within gene sets and the associations that exist between the gene sets and the phenotypes. The biomarker genes of each phenotype were identified by performing WGCNA in the TCGA and GSE33479 datasets, respectively. The soft threshold power was set at 6, the minimum module size was set at 50, and the deep split was set at 2. Venn diagram was used to filter common DEGs shared by the two datasets for later analysis.

### Functional enrichment analysis

The gene probes were first converted into official gene symbols by the online bioinformatics tool in the Database for Annotation, Visualization and Integrated Discovery (DAVID) (https://david.ncifcrf.gov/) [22]. For functional enrichment analysis of the DEGs, the clusterProfiler program was utilized [23], and the optimized *p* value included in the function enrichment analysis was smaller than 0.05.

### PPI network analysis

For the purpose of evaluating PPI network of common DEGs, we used the protein-protein interaction STRING database to retrieve the paired information [24]. Cytoscape software [24] was used to build the PPI network based on the paired relationships between these DEGs. The top ten hub genes in the PPI network were identified using the Cytohubba plugin [25].

### Cell culture and transfection

The NCI-H226 and SKMES1 cell lines used in the experiment were purchased from the Type Culture Storage Center of the Chinese Academy of Sciences (Shanghai, China). Cells were cultured in DMEM supplemented with 10% fetal bovine serum (FBS; Gibco, New York, USA), 100 U/mL of penicillin and 100 μg/mL of streptomycin (Beyotime, Beijing, China) in a humidified incubator containing 5% CO_2_ at 37°C. The overexpression vectors of CHRDL1 and FAM107A were prepared by GenePharma Co., Ltd. (Shanghai, China). Cell transfection was performed using 5 μg plasmid and the PolyFast Transfection Reagent (Yeasen Biotech, Shanghai, China). Functional experiments were performed 48 h after the transfection.

### CCK-8 proliferation assay

The cells were seeded in 96-well plates at the density of 1500 cells per well, and the cells were cultivated in a humidified incubator for indicated duration. At the indicated time point, 15 μL of CCK8 solution (Solarbio, Beijing, China) was added to each well for 3-h incubation. The light absorbance values (OD data at 450 nm) under different conditions were recorded using the Synergy H1 microplate reader (BioTek, Winooski, USA).

### Cell scratch assay

Cells were seeded into 6-well plates and the cells were allowed to reach 80% confluence. A sterile pipette tip was used to create a scratch wound in the central region of the cell monolayer. The floating cells were removed and the fresh medium was replenished. After 24 h, the cell images under different experimental conditions were captured using an inverted light microscope.

### Cell invasion assay

Cells were first re-suspended in the medium without serum. Invasion assays were performed using transwell upper chambers (Corning, New York, USA) coated with Matrigel (BD Biosciences, San Diego, USA). 2.5 × 10^5^ cells in 500 μL serum-free medium were inoculated in the upper chamber. Then the lower chamber was filled with 500 μL of 10% serum-containing medium. After 24 h, cells on the transwell membranes were fixed with ice-cold ethanol for 30 min, and then stained with 0.5% crystal violet (Yeasen Biotech, Shanghai, China) for 15 min. Cells images were taken under an optical microscope.

### Apoptosis analysis

The cells transfected with empty vector or expression vector were harvested and washed twice with PBS. The analysis of cell apoptosis was conducted with the FITC Annexin V Apoptosis Detection Kit (Elabscience, Beijing, China). 1 × 10^6^ cells suspended in the staining buffer were mixed with 5 μL Annexin V-FITC and 1 μL PI reagent for 30-min incubation in the dark. The stained cells were centrifuged at 500 g for 5 min, and then washed with the staining buffer without dyes. After washing, the cells were re-suspended in 500 μL of PBS. Cell apoptosis was detected using BD FACS CantoTM II flow cytometer (BD Biosciences, San Diego, USA).

### Western blot

Total protein sample was extracted using RIPA analysis buffer containing protease inhibitor cocktail (Yeasen Biotech, Shanghai, China). 20 µg of protein sample was subjected to SDS-PAGE gel analysis and blotted to the PVDF membrane. After one hour blocking, the membrane was probed using the following primary antibodies overnight at 4°C: CHRDL1 (H00091851-A01, Amylet Scientific, Beijing, China, 1:1000), FAM107A (HPA055888, Amylet Scientific, Beijing, China, 1:1000), actin (abs100041, Absin, Beijing, China, 1:2000). After washing, the membrane was further labeled with HRP conjugated secondary antibody (1:3000; Absin, Beijing, China, abs20040) for 60 min at ambient temperature. Signal development was conducted using an enhanced chemiluminescence kit (Yeasen Biotech, Shanghai, China). The protein bands were photographed using the Gel-Doc gel imager (Bio-Rad, Philadelphia, USA).

### Statistics

Data analyses were performed using GraphPad Prism 8.0. (GraphPad, New York, USA). Unpaired Student’s *t* test was used to compare the differences between two conditions, and one-way analysis of variance was applied for the comparisons of multiple groups. CCK-8 experimental data at multiple time points were examined using two-way analysis of variance. All the data were reported as the mean ± standard deviation, and the *p* < 0.05 was selected as the threshold for statistical significance.

## Results

### Identification of common DEGs shared by two cohorts of LUSC samples

**Figure 1:**
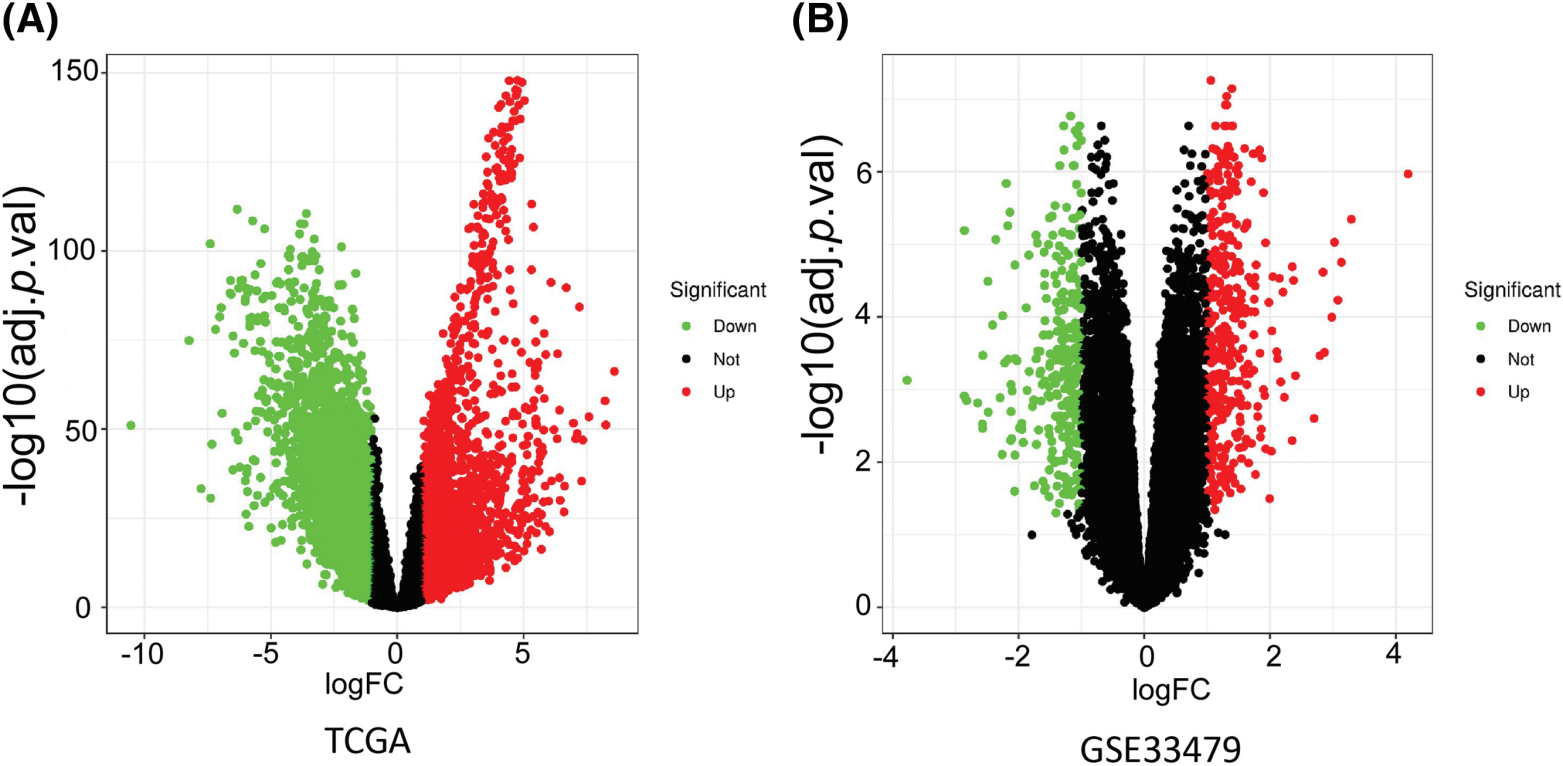
DEG analysis by Limma package in (A) TCGA dataset and (B) GSE33479 dataset. Genes with a fold change (FC) > 2 and an adjusted *p* value less than 0.05 were considered as the DEGs.

The expression data from the TCGA dataset (49 normal samples and 502 LUSC samples) and the GSE33479 dataset (13 normal samples and 14 LUSC samples) were subjected to DEG analysis. The limma package analysis revealed 5131 DEGs from the TCGA-LUSC dataset and 648 DEGs from the GSE33479 dataset, respectively (Figs. 1A and 1B).

**Figure 2:**
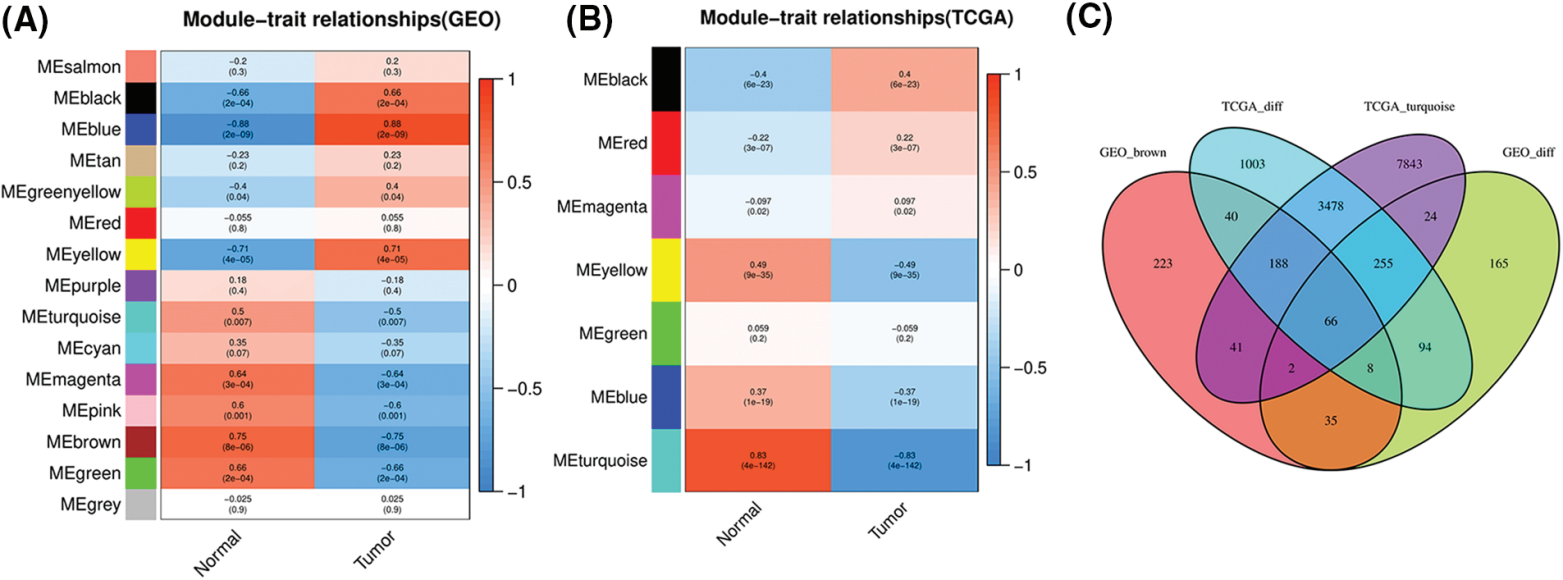
WGCNA analysis of the main gene modules associated with tumor and normal traits in (A) GSE33479 dataset and (B) TCGA-LUSC dataset. (C) Venn diagram showing the intersection of genes in the TGCA_Turquoise modules, genes in GEO_brown module, and the DEGs in GSE33479 and TCGA datasets.

To systematically characterize the gene association patterns between the LUSC and normal samples, we performed WGCNA analysis in each dataset to profile the internal connections of gene sets as the biomarkers of each dataset. 15 gene co-expression modules were identified in the GSE33479 dataset (Fig. 2A), and 7 gene co-expression modules were identified in the TCGA dataset (Fig. 2B). We next screened the most significant connections between the modules and the clinical traits. According to the data, the brown and turquoise modules displayed the strongest and most significant correlations with the tumor-normal traits in the GSE33479 and TCGA dataset, respectively (Figs. 2A and 2B). The brown module consists of 603 genes, and the turquoise module consists of 11,897 genes. According to the intersection of genes in the two modules and the DEGs in GSE33479 and TCGA datasets, Venn diagram showed a total of 66 commonly shared DEGs (Fig. 2C), which were selected as the high-confidence DEGs in the LUSC for further analysis.

### Functional enrichment analyses

**Figure 3:**
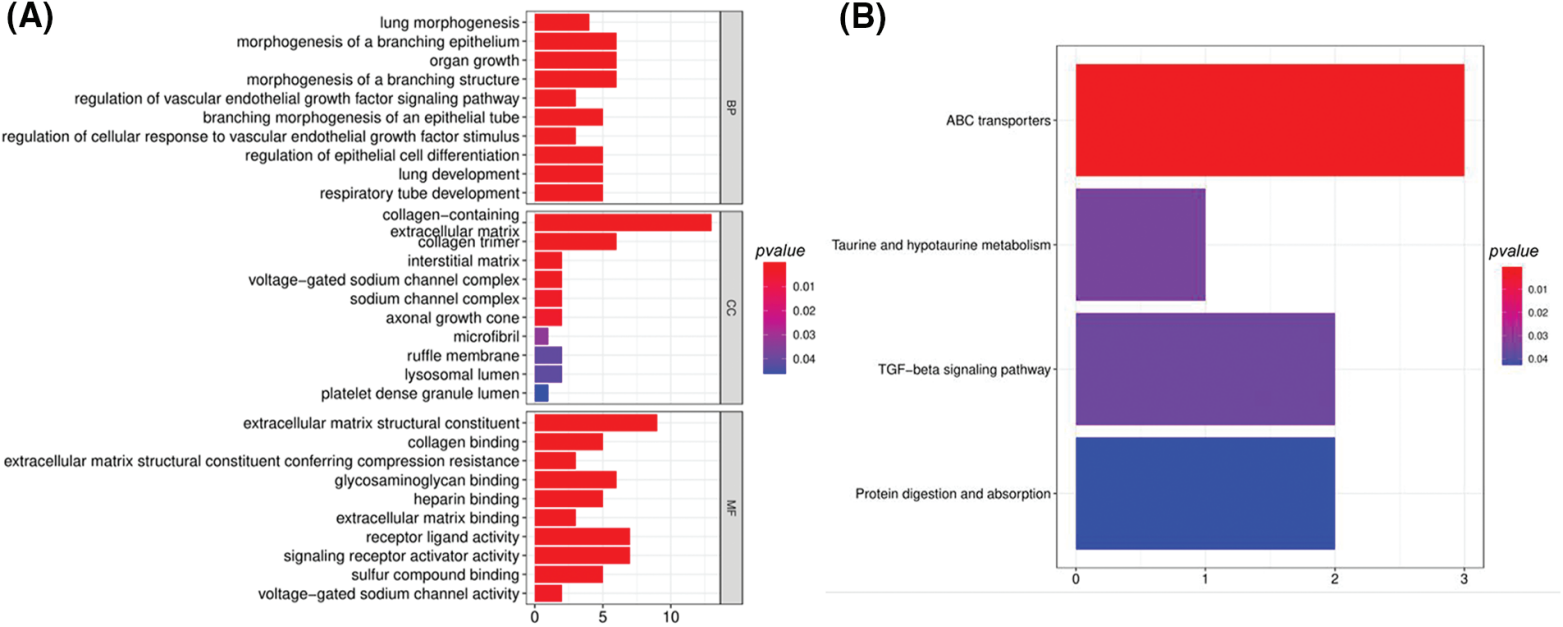
Enrichment analyses of the 66 DEGs by DAVID bioinformatics tool. (A) Gene ontology analysis; (B) analysis with KEGG pathway.

To investigate the biological mechanism of the DEGs, we then performed functional enrichment analyses. The major biological processes (BP) associated with the DEGs included lung morphogenesis, branching epithelium morphogenesis, organ development, and branching structure morphogenesis (Fig. 3A), indicating that those genes may affect lung development. Collagen and extracellular matrix were the top cellular components (CC) associated with the DEGs (Fig. 3A), and the major molecular functions (MF) also revealed extracellular matrix, collagen binding, and glycosaminoglycan binding in the DEGs. After KEGG pathway analyses, taurine transport, protein absorption, typotaurine metabolism and TGF-β signaling pathway were the major signaling processes being altered in the LUSC samples (Fig. 3B).

### Hub gene identification in the PPI network of the DEGs

**Figure 4:**
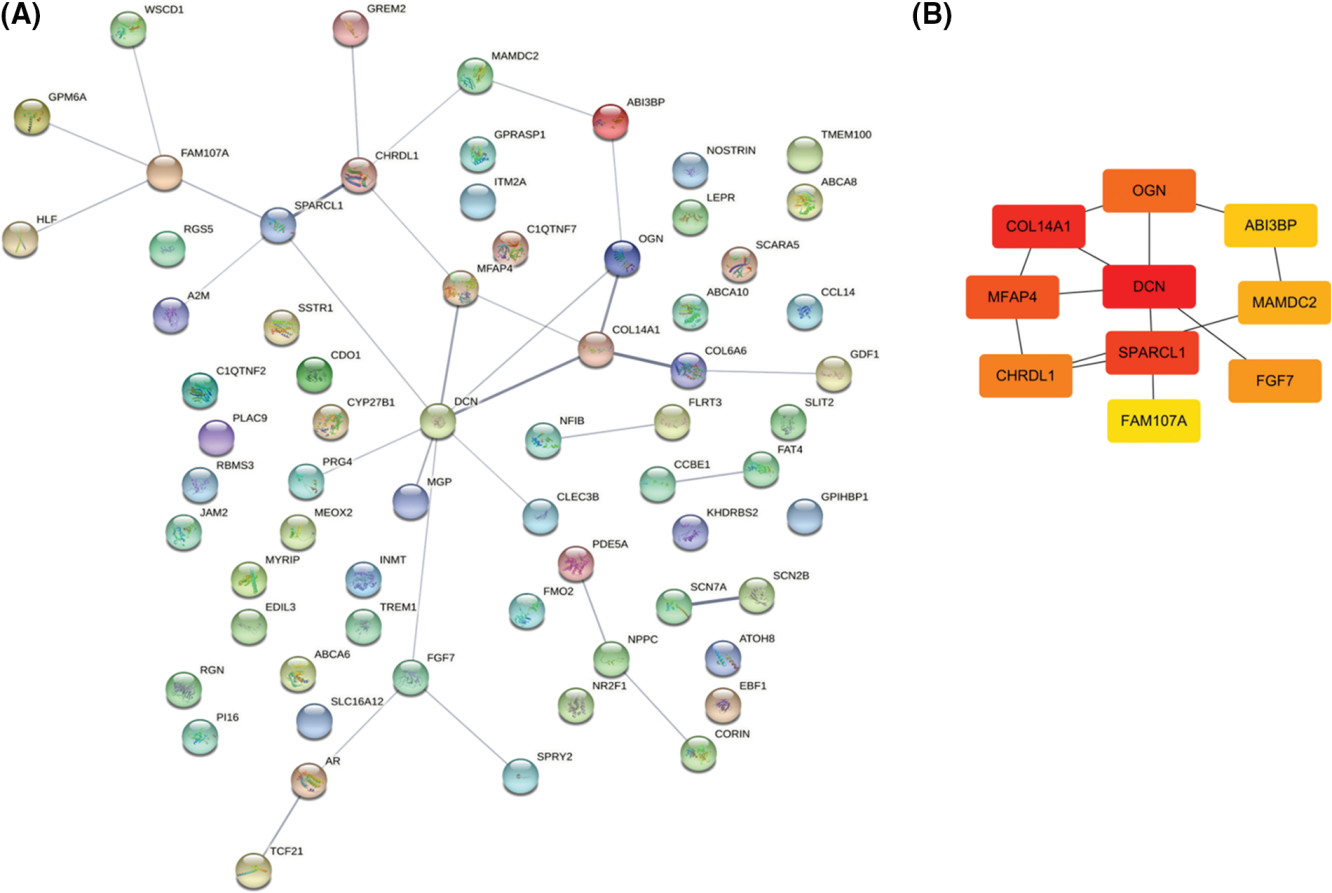
PPI network construction and hub gene identification. (A) A PPI network of 66 DEGs based on the interaction information extracted from the STRING database. (B) Identification of 10 hub genes using the Edge Percolated Component (EPC) algorithm.

**Figure 5:**
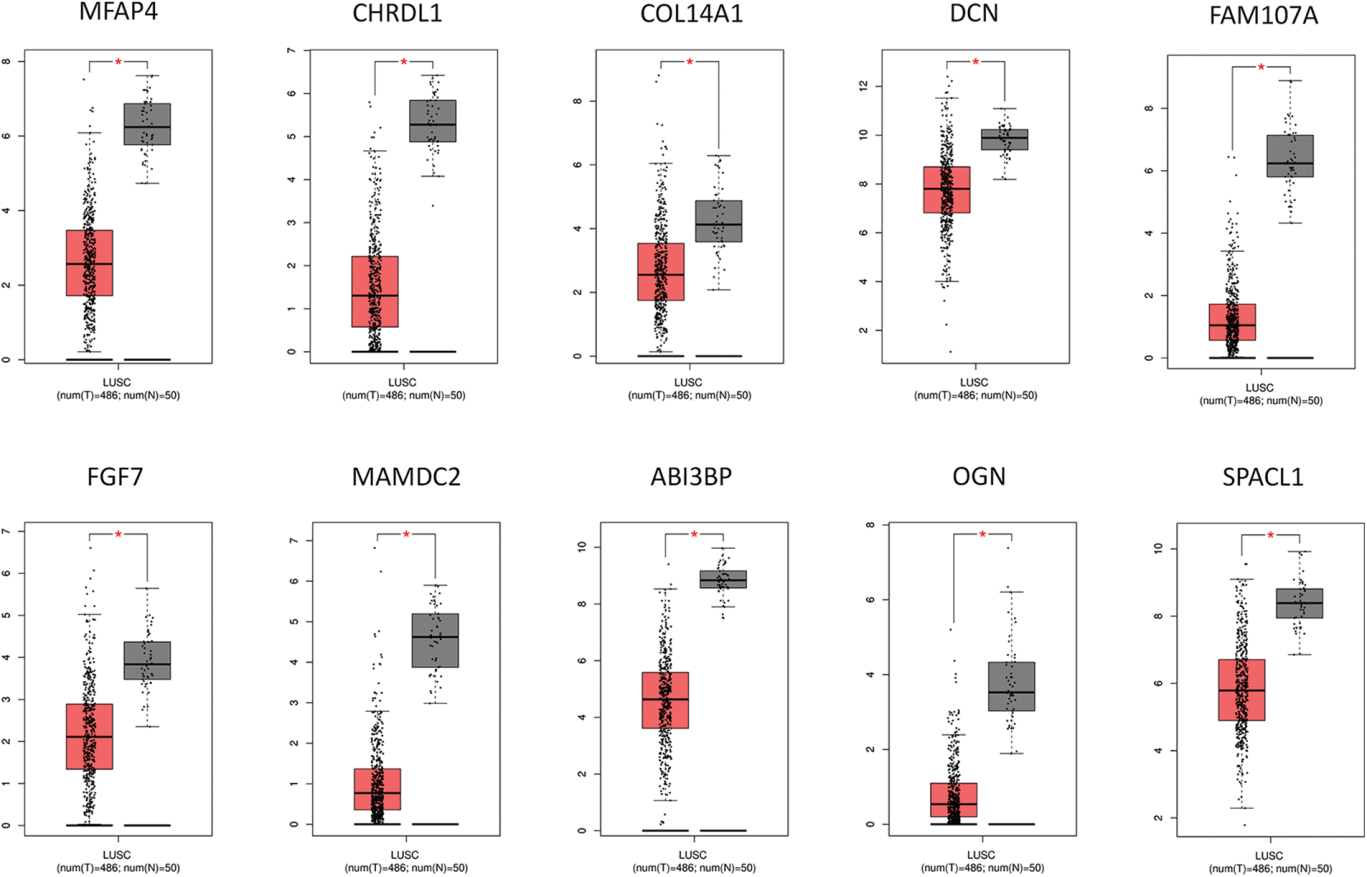
Relative expression levels of the top 10 hub genes in the normal tissues and the LUSC tissues. The normal samples are displayed by the black box, while the LUSC samples are represented by the red box. *p < 0.05.

**Figure 6:**
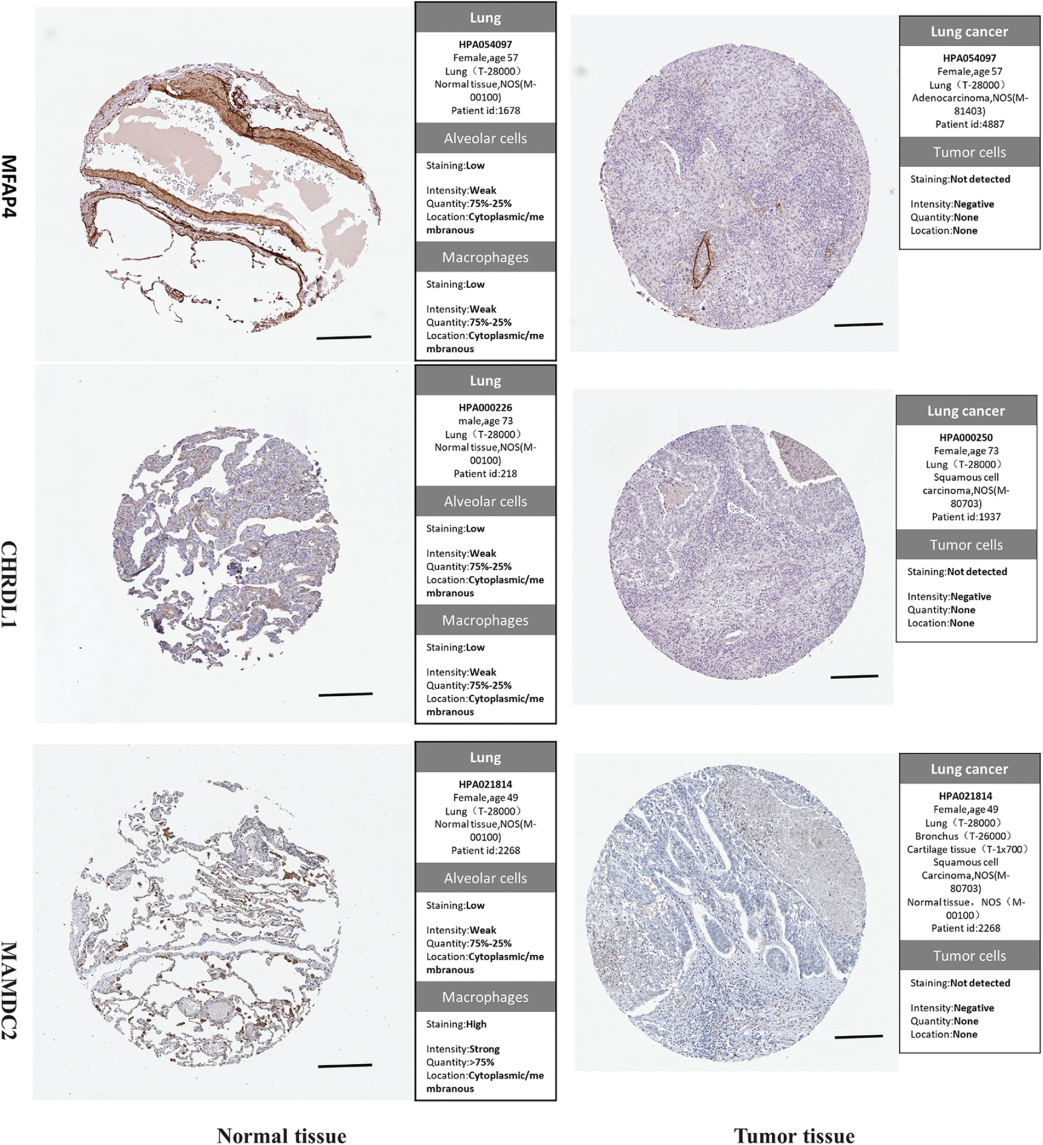
Immunohistochemical staining of the protein levels of MFAP4, CHRDL1, and MAMDC2 in the LUSC and normal tissues from the HPA database. Scale bar: 500 μm.

Then we attempted to establish the PPI network of the DEGs and clarify the central genes involved in LUSC transcriptomic changes using the protein-protein interaction relationship extracted from the STRING database. An interaction network consisting of 66 vertices (nodes) and 31 edges (connections) was constructed (Fig. 4A). To further de-convolute the network and find the key nodes among the interactions, we used the cytoHubba plugin in the Cytoscape software. The Edge Percolated Component analysis reduced the network to 10 nodes which represent the key hub genes, including OGN, ABI3BP, MAMDC2, FGF7, FAM107A, SPARCL1, DCN, COL14A1, MFAP4, and CHRDL1 (Fig. 4B). Notably, all these hub genes showed significant downregulation in the LUSC tumor samples when compared to the normal lung tissues (Fig. 5). The reduced protein levels of MFAP4, CHRDL1, and MAMDC2 were also observed by immunohistochemical staining in the LUSC tissues from the HPA database (Fig. 6). Since tumor suppressor genes tend to be downregulated during the tumor progression, our analyses indicate that there are a panel of tumor suppressor genes with interconnected functions being suppressed in the LUSC tumor samples.

### Functional validation of CHRDL1 and FAM107A as candidate tumor suppressors in LUSC cells

**Figure 7:**
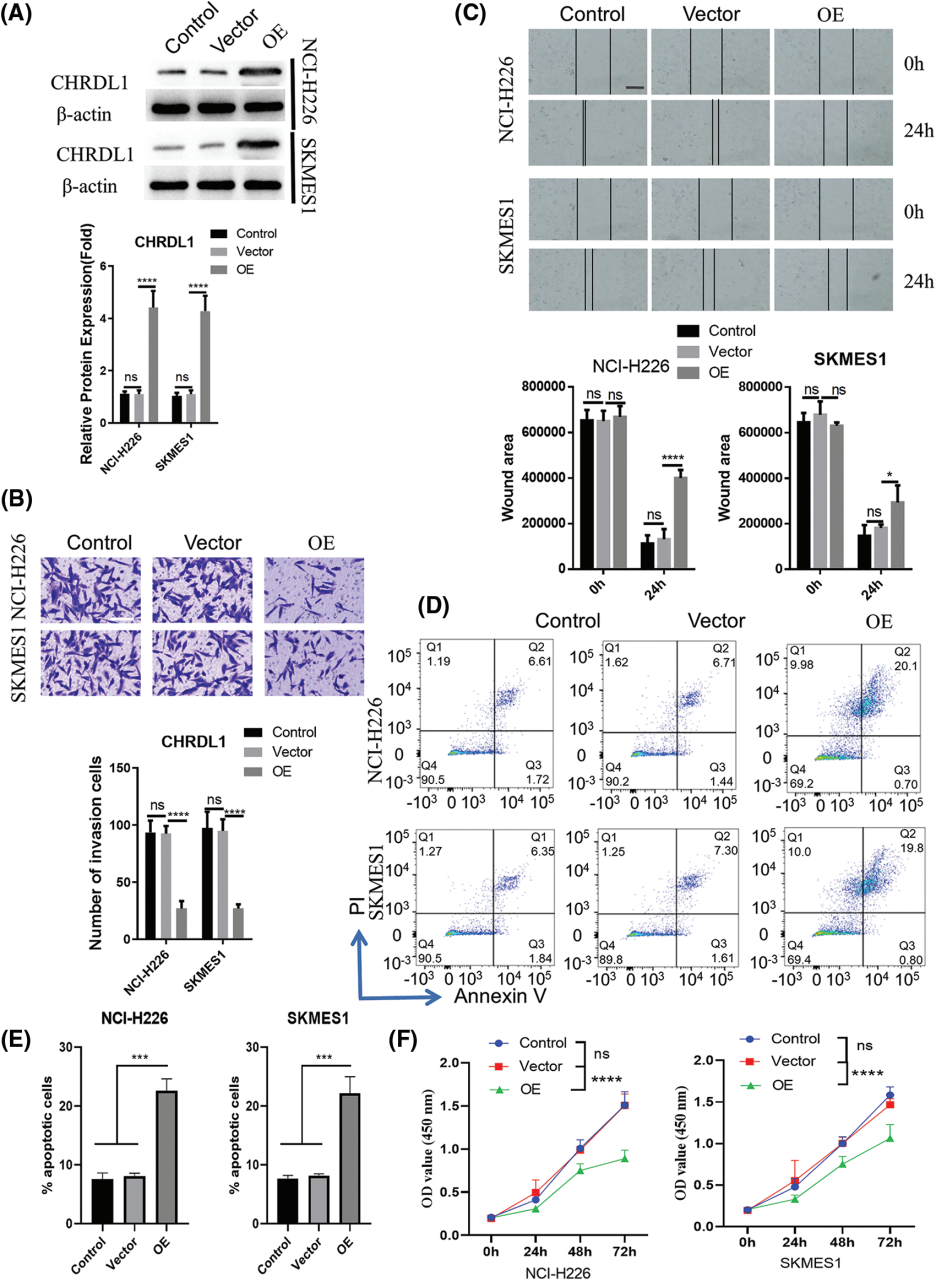
Overexpression of CHRDL1 undermines the malignant phenotype of LUSC cells. NCI-H226 and SKMES1 cells were transfected with empty vector (vector group) or the expression vector of CHRDL1 (OE group). (A) WB was performed to confirm the overexpression of CHRDL1. (B) Transwell invasion assay. (C) Scratch assay. (D, E) Cell apoptosis detection and (F) CCK-8 proliferation assays were performed in above experimental groups. Data were summarized from 3 independent experiments. *p < 0.05; ***p < 0.001; ****p < 0.001. Scale bar: 50 μm in (B) and 100 μm in (C).

**Figure 8:**
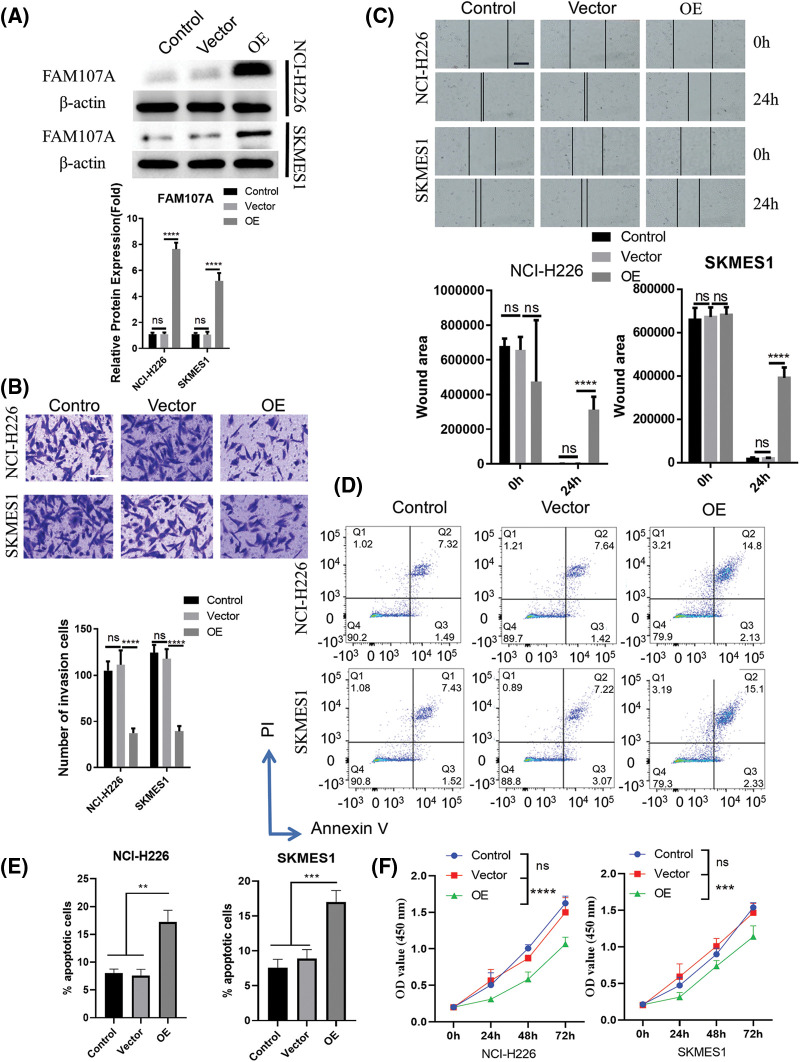
Overexpression of FAM107A undermines the malignant phenotype of LUSC cells. NCI-H226 and SKMES1 cells were transfected with empty vector (vector group) or the expression vector of FAM107A (OE group). (A) WB was performed to confirm the overexpression of FAM107A. (B) Transwell invasion assay. (C) Scratch assay. (D, E) Cell apoptosis detection and (F) CCK-8 proliferation assays were performed in above experimental groups. Data were summarized from 3 independent experiments. **p < 0.01; ***p < 0.001; ****p < 0.001. Scale bar: 50 μm in (B) and 100 μm in (C).

To verify the tumor-suppressive functions of the candidate hub genes, we selected CHRDL1 and FAM107A genes which showed the strongest downregulation in the LUSC samples (Fig. 5). Two Human LUSC cell lines (NCI-H226 and SKMES1) were used as the cell model for gain-of-function analyses. Using empty vector (vector control group) or expression vector for each gene (OE group), we over-expressed each gene in two cell lines and the increased protein contents of CHRDL1 and FAM107A were confirmed by Western blot (Figs. 7A and 8A). Upon the overexpression of CHRDL1 and FAM107A, the invasion ability of NCI-H226 and SKMES1 cells were heavily impaired (Figs. 7B and 8B). The scratch assay also showed the impaired cell migratory capacity after CHRDL1 and FAM107A overexpression (Figs. 7C and 8C). We also performed apoptosis detection by Annexin V and PI staining. The results showed that upon CHRDL1 and FAM107A overexpression, there was a significant increase of apoptotic events in NCI-H226 and SKMES1 cells (Figs. 7D, 7E, 8D and 8E). Additionally, the overexpression of CHRDL1 and FAM107A could also repress the cell growth in NCI-H226 and SKMES1 cells (Figs. 7F and 8F). Together, these data strongly suggest that CHRDL1 and FAM107A may act as tumor suppressors in LUSC cells.

## Discussion

Our study identified commonly downregulated genes in two cohorts of LUSC datasets, and verified the tumor-suppressive functions of two hub genes in the PPI network (CHRDL1 and FAM107A). Overall, our analyses revealed previously unappreciated tumor-suppressor gene network in the LUSC. The inactivation of tumor suppressors are common molecular events in lung cancers. For example, the functional defects of tumor suppressor gene TP53 are one of the most frequent mutations found in lung cancers [26]. Recent work further characterized ATMIN and UBL3 as two potential tumor suppressors in lung cancers [27,28]. It is worth mentioning that different tumor suppressors may be mutated or dysregulated in different stages of tumor progression [29]. Nevertheless, the identification of novel tumor suppressor genes could provide insights into the targeted therapy and diagnosis.

The development of lung cancers is accompanied by the cell de-differentiation, which allows the cells to enter cell cycle and acquire malignant transformation [30,31]. Interestingly, the DEGs identified in two datasets are mostly associated with lung morphogenesis, morphogenesis of a branching epithelium, organ growth, and collagen-containing extracellular matrix, indicating that these genes could be involved in the regulation of the differentiation state of lung epithelial cells. Since these genes were downregulated in LUSC samples, they may be implicated in the maintenance of the differentiation state of lung epithelial cells. Their downregulation may facilitate the de-differentiation of the cancerous cells. This is also evidenced by the observation that the overexpression of CHRDL1 and FAM107A suppressed the malignancy and cell proliferation of LUSC cell lines.

CHRDL1 (chordinlike 1) is located in the X chromosome, encoding a structural glycoprotein (Venotropin) that acts as a binding partner for bone morphogenetic protein 4 (BMP4) [32]. The primary function of CHRDL1 is to antagonize the activity of BMP4 and to promote the differentiation of embryonic cells [33]. In addition, CHRDL1 seems to function as an inhibitor of tumor growth and metastasis in breast cancer, and breast cancer patients with high CHRDL1 expression have better prognosis [32]. Early investigations also suggested that CHRDL1 play a tumor-suppressive role in melanoma [34] and triple-negative breast cancers [35]. Promoter hypermethylation was found to repress CHRDL1 gene in gastric cancer and its downregulation facilitates the proliferation and metastasis of cancer cells [36]. These studies were consistent with our findings, supporting the tumor-suppressive function of CHRDL1. However, the mechanism of CHRDL1 downregulation in LUSC remains to be elucidated.

FAM107A (Family With Sequence Similarity 107 Member A) is an actin-binding protein which regulates the synaptic plasticity by modulating actin filament dynamics [37]. Apart from that, several lines of evidence point to the tumor-suppressive function of FAM107A. In neuroblastoma, FAM107A forms a complex with F-actin to negatively regulate cell cycle progression through suppressing cyclin D1 expression [38]. An early study also revealed the downregulation of FAM107A in renal cell carcinoma [39]. Our study showed the lowered expression of FAM107A in the LUSC samples, and the forced expression of FAM107A not only suppressed the cell proliferation of LUSC cells, but also impaired the cell migration and invasion. Nevertheless, the tumor-suppressive function of FAM107A needs to be further studied in other types of malignancies.

Our study suffers from some limitations. The most significant question is to identify the upstream mechanisms governing the downregulation of these potential tumor suppressor genes. DNA methylation analysis may reveal the epigenetic landscape changes underlying the deregulation of these genes in LUSC cells. Secondly, the detailed molecular mechanisms by which these genes exert tumor-suppressive functions need to be clarified. In the clinical perspective, the association between the expression levels of the candidate tumor-suppressors and the overall survival of cancer patients should be systematically evaluated, which could shed light on the prognostic value of these genes.

To summarize, our study identified a panel of candidate tumor-suppressive genes in LUSC which are functionally connected. Among the candidate genes, we also verified that the forced overexpression of CHRDL1 and FAM107A showed tumor suppressive activity in LUSC cell lines. Future efforts are required to evaluate the clinical significance of these candidates in LUSC patients and unveil the mechanisms underlying their tumor suppressive functions.

## Data Availability

The data generated in the present study may be requested from the corresponding author.
